# Intensity-modulated radiation therapy from 70Gy to 80Gy in prostate cancer: six- year outcomes and predictors of late toxicity

**DOI:** 10.1186/s13014-017-0839-3

**Published:** 2017-06-16

**Authors:** Maria Jolnerovski, Julia Salleron, Véronique Beckendorf, Didier Peiffert, Anne-Sophie Baumann, Valérie Bernier, Sandrine Huger, Vincent Marchesi, Ciprian Chira

**Affiliations:** 1 Departments of Radiation Oncology and Medical Physics, Institut de Cancérologie de Lorraine, 6 Avenue de Bourgogne - CS 30519, 54519 Vandoeuvre-lès-Nancy Cedex, France; 2 Department of Biostatistics, Institut de Cancérologie de Lorraine, 6 Avenue de Bourgogne, 54519 Vandoeuvre-lès-Nancy, France

**Keywords:** Prostate cancer, Intensity-modulated radiation therapy, Rectal and urinary toxicity, Prognostic factors

## Abstract

**Objective:**

To report grade ≥2 overall late rectal and urinary toxicities in patients (pts) with prostate cancer treated by intensity-modulated radiotherapy (IMRT) at 3 dose-levels. Identify predictors of radiation toxicity and report biochemical progression free survival (bPFS).

**Methods:**

A total of 277 pts were treated with 70Gy (10.8%), 74Gy (63.9%) and 80 Gy (25.3%) using IMRT without pelvic irradiation were analyzed. Short or long-course androgen deprivation therapy (ADT) was allowed in 46.1% of pts. The toxicity was described using the Common Terminology Criteria for Adverse Events (CTCAE) v4.0 scale. Cox regression models addressed demographics, disease and dosimetry characteristics as potential predictors of late grade ≥2 toxicity after adjusting for other modifying factors.

**Results:**

The median follow-up was 77 months (range 15; 150). There was no grade ≥4 toxicity. The 5-year cumulative rate of grade ≥2 late rectal and urinary toxicities was 6.3% (95% CI = 3.8%; 10.3%) and 25.3% (95% CI = 19.8%; 31.8%) respectively. In multivariate analysis, only the dose (80Gy vs 74 and 70Gy) was found to increase the risk of rectal toxicity (HR = 2.96 [1.07; 8.20]). For pts receiving 74 Gy, International Prostate Symptom Score (IPSS) at baseline ≥8 (HR = 2.40 [1.08; 5.35]) and dose ≥73Gy delivered in more than 2% of bladder (D2%) were found to be predictors of bladder toxicity (HR = 3.29 [1.36; 7.98]). The 5–year biochemical relapse free survival was 81.0% [74.5%; 86.0%] in the entire population, 97.5% [83.5%; 99.6%] in the low risk group, 84.9% [76.7%; 90.3%] in the intermediate risk group and 66.4% [51.8%; 77.4%] in the high-risk group. D’Amico low (HR = 0.09 [0.01; 0.69]) and intermediate risk groups (HR = 0.50 [0.28; 0.88]) as well as PSA nadir ≥0.2 ng/ml (HR = 1.79 [1.01; 3.21]) were predictive of biochemical relapse.

**Conclusions:**

The rate of late rectal toxicity increased with higher doses, while Dmax ≥74Gy, D2% ≥ 73Gy for bladder wall and baseline IPSS ≥8 increased late urinary toxicity.

## Introduction

External beam radiation therapy is commonly used to treat with curative intent prostate cancer. During the past two decades, 7 randomized trials and one meta-analysis have shown improvements of 10 to 20% in biological progression-free survival rates (bPFS) and freedom from metastatic disease (in D’Amico high risk patients) by using dose-escalated radiotherapy [1–8]. However, increasing the dose carries a potential risk of severe rectal and urinary side effects [9–12], especially when former conventional radiation techniques like three-dimensional conformal radiotherapy (3D-CRT) are used.

Nowadays, the use of modern intensity-modulated radiotherapy (IMRT) has shown to significantly reduce acute toxicity rates compared with what has been observed with 3D-CRT, and biochemical control rates are promising [13]. However, mature data on late toxicity and biochemical outcome is still lacking even if earlier studies of prostate IMRT are beginning to report longer follow-up.

The primary objective of our study was to report outcomes, including grade ≥2 overall late rectal and urinary toxicity and biochemical control rates in pts treated with IMRT to 70Gy, 74Gy and 80Gy. We also aimed to identify potential clinical as well as dosimetric predictors of late toxicity in pts receiving moderate dose IMRT (74Gy-group).

## Materials and methods

We retrospectively reviewed medical and dosimetry records of pts who received radical IMRT in our center for a prostate cancer. Eligibility criteria included: untreated histologically confirmed adenocarcinoma of the prostate, stage T1c-T4 N0 M0 according to the 1992 American Joint Committee on Cancer staging system. Pts were stratified by prognostic risk groups based on D’Amico criteria [14]. Pts with positive pelvic lymphadenectomy or radiological positives nodes were excluded. Androgen deprivation therapy (ADT) was allowed in D’Amico intermediate and high-risk pts. The following data were extracted from medical records: age, surgical history (prior abdominal surgery, transurethral resection of prostate), anticoagulant or antiplatelet treatment, diabetes, hypertension and coronary insufficiency as well as tumor characteristics (T stage, Gleason score, pretreatment PSA). Dosimetric parameters were extracted for pts receiving 74Gy and are detailed in statistical methods.

The CTCAE v.4 criteria was used to evaluate late toxicity (≥6 months from the start of treatment) for all patients. Toxicity scores were recalculated for patients treated before publication of CTCAE v4.0 scale. The radiation oncologist and urologist recorded peak toxicity grades every 6 months for at least 5 consecutive years.

The RTOG-ASTRO Phoenix Consensus definition of biochemical relapse (nadir + 2ng/ml) was applied for pts treated after 2006 [15]. For all others, 3 consecutive PSA rises after a nadir with the date of failure as the point halfway between the nadir date and the first rise or any rise great enough to provoke initiation of therapy was also considered as a biochemical failure [16].

### Treatment planning

All pts underwent treatment planning computed tomography (CT) (Philipps Brilliance 4.0) with a slice thickness of 3 mm. Images were then transferred to the treatment planning system (Eclipse Varian Medical Systems v7-10). Pts were asked to empty their rectum and bladder 1 h prior to the treatment planning CT scan and daily treatment and invited to drink water in order to have 100–400 ml in the bladder. Bladder filling was verified with a portable bladder-scan. Target and organ at risk delineations followed the recommendations of the French Study Group on Urogenital Tumors (GETUG) [3]. Briefly, the planning target volume (PTV) was calculated by adding a 10-mm margin to the prostate and seminal vesicles in all directions except posteriorly (5mm). Pts were treated in the supine position with COMBIFIX™ device (CIVCO Medical Solutions) for pelvic setup. The radiation dose was escalated over time starting from 70Gy to 74Gy and finally to 80Gy. The dose was prescribed and normalized so that the PTV was included within 95% isodose line. The dose per fraction was specified at the International Commission on Radiation Units and Measurements point. All pts were treated with 10 and 23 MV photons using 5 static IMRT sliding-windows beams at angles of (IEC-scale) 0°, 80°, 130°, 230°, 280°, respectively. Dose constraints used for validation of treatment planning are detailed in Table 1. Two hundred and eighteen pts (78.7%) were treated with a simultaneous integrated boost (SIB), which delivered 2Gy/fraction to the prostate and 1.4Gy to 1.6Gy/fraction to the seminal vesicles. All others were treated in 2 phases: phase I, 46 Gy in 2 Gy/fraction, 5 times/week to the PTV1 (prostate and seminal vesicles), while in phase 2, pts received 24 or 34 Gy to the prostate alone (PTV2). IMRT was delivered with online imaging guidance at day 1, 2, 3 and then weekly. This study was approved by our Institutional Review Board and Ethics Committee and conducted in accordance with the Declaration of Helsinki, Good Clinical Practice and French regulatory requirements.

**Table 1:** Dose constraints

Dose	70Gy arm	74Gy arm	80Gy arm
Rectal wall	V45_Gy_ ≤40%	V68_Gy_ < 25%	V50_Gy_ < 46%
	V65_Gy_ ≤25%	V45_Gy_ < 45%	V72_Gy_ < 25%
	D2% ≤70Gy	D2% ≤74Gy	Dmax ≤76Gy
Bladder wall	V50_Gy_ ≤ 35%	V50_Gy_ <40%	V70_Gy_ ≤ 50%
	D2% ≤ 70Gy	V65_Gy_ <25%	Dmax ≤ 80Gy
		D2% ≤ 74Gy	

*Abbreviations*: *V* volume, *D* Dose, *Ex.: V65Gy ≤25%* 25% of rectal wall volume receiving no more than 65 Gy, *Dmax* maximum point dose to an organ, *D2%* Dnearmax

### Statistical methods

Cumulative incidence of bladder toxicity was described with the Kaplan Meier method. The event of interest was overall late urinary toxicity with a grade ≥2. The follow-up time was censored at 84 months for pts with no events. For pts with loco-regional or metastatic progression, the observation of late toxicity was censored at the moment of relapse.

Potential prognostic factors of urinary toxicity were assessed with bivariate Cox proportional hazard model (CPHM). Age was dichotomized according to its median value and IPSS according to a threshold of ≥8 [17]. Parameters with a *p*-value less than 0.2 and age were included into a multivariate CPHM with backward selection. Our dosimetry constraints on bladder wall (Table 1) were tested by a bivariate analysis in the whole population. Patients “were stratified” according to deviations from DVH constraints (Table 1). For example, group 0 = full compliance with all DHV constraints; group 1 = minor or major deviation from 1st DHV constraint (V50 ≤ 35% for 70Gy-arm, V50 ≤ 40% for 74-arm, V70 ≤ 50% for 80-arm); group 2 = minor or major deviation from 2nd DHV constraint, or a combination of non-compliance to all constraints. Our bivariate model aimed to test if non-compliance to one or all of the constraints was predictive of grade ≥2 toxicity. A second analysis of dosimetry data was only performed in the 74-Gy subgroup. The 3 constraints detailed in Table 1 as well as close values to these given constraints were tested as prognostic factors for bladder toxicity by a bivariate CPHM. In case of significant result, the threshold that maximized the log-likelihood function was chosen in order to dichotomize the parameter.

Cumulative incidence of rectal toxicity was described with the Kaplan Meier method and bivariate and multivariate CPHM as described for the urinary toxicity. For dosimetry analysis, our constraints on rectal wall (Table 1) were tested in the whole population and the in the 74Gy-arm subgroup by the same bivariate CHPM model as described above for urinary toxicity.

The bPFS survival was also described by Kaplan Meier method. The event of interest was a biochemical relapse or death (all causes) whichever occurred first. Other events were ignored. Patients alive and free of biochemical relapse were censored at 84 months. Potential prognostic factors were assessed with bivariate CPHM. Threshold for PSA nadir was assessed according to the maximization of the log-likelihood function in CPHM. Parameters with a p-value less than 0.2 and the age were included into a multivariate CPHM with backward selection. Note that stage, Gleason and PSA at baseline were not selected in this procedure as these are strongly linked to D’amico Risk group. The stability of all multivariate CPHM was investigated using a bootstrap resampling method [18].

The median PSA nadir (PSAn) and time to PSA nadir (tPSAn) were compared between radiation doses with a Kruskal Wallis test after testing the normality of the distribution by a Shapiro-Wilk test. For patients without ADT receiving 74Gy, tPSAn was tested as a prognostic factor of biochemical relapse free survival in a bivariate CPHM and dichotomized according to the maximization of the log-likelihood function in CPHM. Multivariate CPHM was performed in order to adjust the result on the prognostic factors found in the entire population.

Cumulative rate of locoregional and metastatic failure was estimated by the Kaplan Meier method. All these results were expressed with hazard ratio and 95% confidence interval. A p-value less than 0.05 was considered statistically significant. All analyses were performed with SAS software, version 9.2 (SAS, Cary, NC, USA).

## Results

Between June 2000 and December 2010, two hundred seventy-seven pts were treated by IMRT for a prostate cancer in our center. The median age was 69 years (range 51; 79). The median follow-up was 77 months (15; 150) for pts alive at last follow-up. All pts completed the planned treatment, except one patient who stopped treatment at 78 Gy and was analyzed in the 80Gy-group. Two patients were excluded from the morbidity analysis because follow-up was shorter than 6 months.

One hundred and twenty-eight pts (46.1%) had undergone ADT (≤ 6 months for 70 pts and ≥6 months for 57 pts). The median treatment duration was 57 days (46; 76). The dose planning data from 237 pts was available for analysis. Pretreatment patients’ characteristics are listed in Table 2.

**Table 2:** Patients characteristics

		RTH dose prescribed			
Characteristics	All (*n* = 277,100%)	70Gy (*n* = 30, 10.8%)	74Gy (*n* = 177, 63.9%)	80Gy (*n* = 70, 25.3%)	*p*-value
Age (years)	69 (51–79)	70 (56;79)	71 (51;79)	68.5 (51;76)	0.03
Follow up (months)	53.1 (3.4–150)	48.4 (3.9;94.8)	47.1 (3.4;150.2)	75.6 (7.8;131.7)	<0.01
PSA level (ng/ml)					
<10	166 (59.9%)	26 (86.7%)	105 (59.4%)	35 (50%)	<0.01
10–20	79 (28.5%)	3 (10%)	19 (10.7%)	10 (14.3%)	
>20	32 (11.6%)	1 (3.3%)	53 (29.9%)	25 (35.7%)	
Gleason					
6	88 (32.1%)	23 (76.7%)	46 (26.3%)	19 (27.5%)	<0.01
7 (3 + 4)	84 (30.7%)	4 (13.3%)	52 (29.7%)	28 (40.6%)	
7 (4 + 3)	60 (21.9%)	1 (3.3%)	47 (26.9%)	12 (17.4%)	
8–10	42 (15.3%)	2 (6.7%)	30 (17.1%)	10 (14.5%)	
Tumor stage					
T1-T2a	178 (64.3%)	25 (83.3%)	110 (62.2%)	43 (61.4%)	0.15
T2b	44 (15.9%)	3 (10%)	27 (15.3%)	14 (20%)	
T2c-T4	55 (19.9%)	2 (6.7%)	40 (22.5%)	13 (18.6%)	
D’Amico risk group					
Low risk	41 (14.8%)	20 (66.7%)	20 (11.3%)	1 (1.4%)	<0.01
Intermediate risk	161 (58.1%)	7 (23.3%)	103 (58.2%)	51 (72.9%)	
High risk	75 (27.1%)	3 (10%)	54 (30.5%)	18 (25.7%)	
Androgen deprivation					
No	149 (53.9%)	23 (76.7%)	90 (50.9%)	36 (51.4%)	0.03
Yes	128 (46.1%)	7 (23.3%)	87 (49.1%)	34 (48.6%)	
Short (< 6 months)	70 (25.4%)	4 (13.3%)	38 (21.7%)	28 (40%)	<0.01
Long (> 6 months)	57 (20.7%)	3 (10%)	48 (27.4%)	6 (8.6%)	
IPSS baseline					
0–7	236 (85.2%)	24 (80%)	154 (87%)	58 (82.9%)	0.49
≥8	41 (14.8%)	6 (20%)	23 (13%)	12 (17.1%)	
Diabetes					
Yes	49 (17.9%)	5 (16.7%)	33 (18.9%)	11 (15.9%)	0.85
Coronary disease					
Yes	46 (16.9%)	5 (18.5%)	31 (17.6%)	10 (14.5%)	0.82
High blood pressure					
Yes	151 (54.9%)	18 (60%)	98 (55.7%)	35 (50.7%)	0.66
Surgery					
Abdominal	29 (10.5%)	6 (20%)	17 (9.6%)	6 (8.6%)	0.19
Pelvic	86 (31.1%)	2 (6.7%)	46 (26%)	38 (54.3%)	<0.01
Other	46 (16.7%)	7 (23.3%)	28 (15.8%)	11 (15.7%)	0.57
Transurethral resection of prostate					
Yes	38 (13.8%)	20% (6)	14.29% (25)	10% (7)	0.40
Alpha blocker treatment at D-1 RTH					
Yes	57 (20.7%)	6 (20%)	44 (25.1%)	11 (15.7%)	0.26
Anticoagulant or antiplatelet treatment					
Yes	112 (40.7%)	11 (36.7%)	78 (44.6%)	23 (32.9%)	0.21

Qualitative parameters are described with frequency and percentage; quantitative parameters with median and range

*Abbreviations*: *PSA* prostate specific antigen, *risk group* D’Amico classification, *IPSS* International Prostate Score Symptom, *RTH* radiotherapy, *D-1* day one of treatment

I. **Late toxicity**
  - **Rectal**
    There was no grade ≥4 toxicity. The cumulative rate of overall grade ≥2 late toxicity at 5 years was 6.3% (95% CI = 3.8–10.3). Grade 2 was experienced by 9 pts and consisted of minor rectal bleeding, diarrhea and rectal pain. Six pts experienced grade 3 toxicity namely rectal bleeding that needed invasive intervention. All grade 2–3 rectal side effects were observed in pts with no ADT or ≤6 months ADT.
    Bivariate analyses of prognostic factors are presented in Table 3. Doses of 74Gy versus 70Gy had no statistically significant impact on rectal toxicity. In multivariate analysis only the dose of 80 Gy, compared to 70 and 74Gy, was significantly associated with increased toxicity (HR = 2.96 [1.07; 8.20], *p* = 0.04) (Table 3).

**Table 3:** Rectal toxicity pronostic factors

Characteristics	Bivariate analysis		Multivariate analysis	
	HR and 95% CI	*p*-value	HR and 95% CI	*p*-value
Age^a^				
≤70	1			
>70	0.44 [0.15; 1.28]	0.13	-	-
Stage				
T1c + 2a	1			
T2b	1.68 [0.52; 5.46]	0.39		
T2c-T4	0.69 [0.15; 3.19]	0.63		
Diabetes				
No	1			
Yes	1.84 [0.59; 5.80]	0.30		
HBP				
No	1			
Yes	0.55 [0.20; 1.53]	0.25		
Pelvic surgery				
No	1			
Yes	0.89 [0.32; 2.52]	0.84		
Previous TURP				
No	1			
Yes	0.98 [0.22; 4.36]	0.98		
ADT^a^				
No	2.46 [0.78;7.74]	0.12		
Yes	1			
Anticoagulation treatment^a^				
No	1			
Yes	1.30 [0.47; 3.59]	0.61	-	-
RTH total dose				
70 Gy	1			
74 Gy	1.06 [0.13;8.82]	0.96		
80 Gy	3.12 [0.39;24.98]	0.28		
RTH total dose^a^				
70Gy-74Gy	1			
80 Gy	2.96 [1.07; 8.20]	0.04	2.96 [1.07; 8.20]	0.04

*Abbreviations*: *HR* hazard ratio, *CI 95%* 95% confidence interval, *HBP* high blood pressure, *TURP* transurethral prostate resection, *ADT* androgen deprivation therapy, *anticoagulation treatment* anticoagulation or antiplatelet treatment, *RTH* radiotherapy

^a^Parameters included in backward multivariate analysis
    The non-compliance of IMRT treatment plans to our constraints on rectal wall (Table 1) was not a significant predictor of rectal toxicity (data not shown).
  - **Urinary**
    There was no ≥4 grade toxicity. The 5-year cumulative incidence of overall grade ≥2 overall toxicity was 25.3% (95% CI = 19.8–31.8). Only two pts had grade 3 toxicity namely dysuria and pollakiuria. The predominant grade 2 toxicity was dysuria (19 pts), pollakiuria (16 pts), nycturia (12pts) and urgency (6 pts). In multivariate analysis, only the IPSS at baseline ≥8 was associated with an increased risk of toxicity (HR = 2.43 [1.37; 4.31], *p* < 0.01) (Table 4).

**Table 4:** Urinary toxicity prognostic factors

Characteristics	Bivariate analysis		Multivariate analysis	
	HR and 95% CI	*p*-value	HR and 95% CI	*p*-value
Age^a^				
≤70	1			
>70	1.15 [0.69; 1.91]	0.59	-	-
Stage				
T1c + 2a	1			
T2b	0.86 [0.40; 1.85]	0.70		
T2c-T4	1.27 [0.70; 2.30]	0.43		
Diabetes^a^				
No	1			
Yes	1.96 [1.09; 3.54]	0.02	-	-
HBP				
No	1			
Yes	1.02 [0.61; 1.70]	0.95		
Pelvic surgery				
No	1			
Yes	1.18 [0.59; 2.0]	0.53		
Previous TURP^a^				
No	1			
Yes	0.29 [0.09; 0.94]	0.04	-	-
Androgen deprivation				
No	1			
<6m	1.28 [0.71; 2.30]	0.41		
>6m	1.15 [0.60; 2.21]	0.67	-	-
Anticoagulation treatment^a^				
No	1			
Yes	0.71 [0.41; 1.21]	0.20	-	-
RTH total dose				
70 Gy	1			
74 Gy	0.71 [0.33;1.54]	0.38		
80 Gy	0.84 [0.37;1.92]	0.70		
RTH total dose^a^				
70Gy-74Gy	1			
80 Gy	1.1 [0.64; 1.92]	0.70	-	-
IPSS baseline^a^				
0–7	1			
≥8	2.43 [1.37; 4.31]	<0.01	2.43 [1.37; 4.31]	<0.01

*Abbreviations*: *HR* hazard ratio, *95% CI* 95% confidence interval, *HBP* high blood pressure, *TURP* transurethral prostate resection, *anticoagulation treatment* anticoagulation or antiplatelet treatment, *RTH* radiotherapy, *IPSS* international prostate score symptom

^a^Parameters included in backward multivariate analysis
    The non-compliance of IMRT treatment plans to our constraints on bladder wall (Table 1) was not a significant predictor of bladder toxicity (data not shown). In 74-Gy group, Dmax ≥ 74Gy (HR = 2.08 [1.04; 4.16], *p* = 0.04) and D2% ≥ 73Gy (HR =3.32 [1.37; 8.04], *p* < 0.01) were prognostic of grade ≥ 2 late toxicity in bivariate analyses. The five-year cumulative toxicity grade ≥2 was 30.6% [19.1%; 46.9%] in pts with Dmax ≥ 74Gy (vs 18.3% [11.9%; 27.7%]) and 31.7% [22.7%; 43.2%] in patients with D2% ≥ 73Gy (vs 7.6% [3.2%; 17.3%]). Only D2% ≥ 73Gy (HR = 3.29 [1.36; 7.98], *p* < 0.01) was significantly associated with bladder toxicity after adjusting for IPSS at baseline ≥ 8 (HR = 2.40 [1.08; 5.35], *p* = 0.03) the only prognostic factor in the all population.
    Nine pts (3.3%) were staged T3b before initiation of radiation therapy. Grade 1 diarrhea was seen in 2 of these pts while grade 1 et 2 dysuria, nocturia and hematuria toxicity was recorded in 3 pts. One patient (0.4%) was staged T4 due to suspicion of rectum invasion on MRI. He was treated with 74Gy and ADT. We found a peak grade 1 rectal toxicity for this patient. According to medical records he developed minor uncomfortable anal leaking 52 months from the end of radiation.
  - **Tumor control**
  - **Biochemical control:**
    The 5–year biochemical relapse free survival was 81.0% (95% CI = 74.5%; 86.0%) in the entire population, 97.5% (95% CI = 83.5; 99.6) in the low risk group, 84.9% (95% CI = 76.7; 90.3) in the intermediate risk group and 66.4% (95% CI = 51.8%; 77.4%) in the high-risk group.
    Among 43 local relapses, 30 were treated by ADT, 3 by chemotherapy and 1 by high intensity frequency ultrasound (HIFU). Eight patients died without local relapse.
    In multivariate analysis, the D’Amico low-risk group (HR = 0.09 [0.01; 0.69], *p* = 0.02), intermediate risk group (HR = 0.50 [0.28; 0.88], *p* = 0.02) compared to high risk group and PSA nadir ≥0.2 ng/ml (HR = 1.79 [1.01; 3.21], *p* = 0.04) were significantly associated with biochemical relapse free survival (Table 5).

**Table 5:** Biochemical failure prognostic factors

Characteristics	Bivariate analysis		Multivariate analysis	
	HR and 95% CI	*p*-value	HR and 95% CI	*p*-value
Age^a^				
≤70	1			
>70	1.21 [0.69; 2.13]	0.49	-	-
Stage				
T1c + 2a	1			
T2b	1.88 [0.91; 3.89]	0.09		
T2c-T4	2.11 [1.11; 4.03]	0.02		
D’Amico Risk group^a^				
Low	0.11 [0.01; 0.79]	0.03	0.09 [0.01; 0.69]	0.02
Intermediate	0.57 [0.32; 0.99]	0.04	0.49 [0.28; 0.88]	0.02
High	1			
% Positive biopsy^a^				
≥50%	1.72 [0.98; 3.05]	0.06	-	-
<50	1			
Gleason				
6	1			
3 + 4	2.39 [0.92; 6.24]	0.07		
4 + 3	5.04 [1.99; 12.72]	<0.01		
8–10	3.56 [1.32; 9.65]	0.01		
PSA baseline				
<10	1			
10–20	1.03 [0.53; 2]	0.92		
>20	2.71 [1.38; 5.34]	<0.01		
Androgen deprivation				
No	1			
<6m	1.15 [0.59; 2.25]	0.66		
≥6m	1.33 [0.66; 2.69]	0.42		
RTH total dose				
70 Gy	1			
74 Gy	2.56 [0.61;10.70]	0.20		
80 Gy	1.59 [0.36;7.05)	0.54		
RTH total dose^a^				
70-74Gy	1			
80 Gy	0.67 [0.36; 1.25]	0.21	-	-
Nadir^a^				
<0.2	1			
≥0.2	1.52 [0.86; 2.68]	0.15	1.79 [1.01; 3.21]	0.04

*Abbreviations*: *HR* hazard ratio, *95% CI* 95% confidence interval, *PSA* prostate specific antigen, *RTH* radiation therapy

^a^Parameters included in backward multivariate analysis
  - **Time to nadir (for the 149 pts without ADT):**
    The tPSAn was 32 months (range 4; 65) for 70 Gy, 24 months (3; 76) for 74 Gy and 38 months (2; 116) for 80 Gy (*p* = 0.02).
    For patients without ADT (90 pts) in the 74-Gy arm, the tPSAn ≤6 months was prognostic of biochemical relapse in bivariate analysis (HR = 6.52 [1.65; 25.74], *p* < 0.01). After adjustment for D’Amico risk group and PSA nadir ≥0.2 ng/ml (the prognostic factors of biochemical relapse in the entire population), the tPSAn ≤6 months remained statistically significant (HR = 5.62 [1.31; 24.04], *p < 0.01*).
  - **Loco regional and metastatic failure:**
    The 5-year cumulative incidence of locoregional failure was 9.2% (95% CI = 5.7%; 14.7%). At the last follow-up, twenty-four pts experienced a locoregional failure: 9 of them had a prostate and/or seminal vesicles relapse and 15 had a lymph pelvic node relapse. Twenty of them were treated by either ADT (18 pts), radiotherapy (1 pt) or High Intensity Focuse Ultrasound (1 pt). The median time from nadir to relapse was 34 months (range 5; 81).
    The 5-year cumulative incidence of metastatic failure was 7.3% (95% CI = 4.3%;12.3%). Eighteen pts had metastatic disease at the last of follow-up: 14 pts had bone metastasis and 5 had lymph nodes metastasis outside the pelvis. Treatment included ADT for 13 pts, chemotherapy (2 pts), radiotherapy (1pt) and abiraterone acetate (1 pt). The median time from nadir to relapse was 21 months (5; 103).

## Discussion

Our results suggest that radiation doses of 80 Gy are associated with a greater likelihood of long-term overall grade ≥2 rectal toxicity but failed to identify other predictors. However, rates of late rectal toxicity, grade 3 CTCAE in particular, were low with IMRT (6.2%). Concerning urinary toxicity, moderate to severe baseline IPSS (≥ 8) worsened radiation-induced toxicity. Dosimetric parameters, especially D2% ≥73Gy, may be used as a surrogate predictor of late urinary toxicity in moderate escalation IMRT (74Gy). To the best of our knowledge, our report is the first to compare 70Gy, 74Gy and 80Gy with IMRT in prostate cancer.

We believe that our findings are an important addition to the previously published data on prostate IMRT, and this for several reasons.

Firstly, our study reports overall late toxicity outcomes and biochemical control rates from a comparison between 3 radiation doses levels, the lower and upper bounds being set at 70Gy and 80 respectively. Indeed, it is now well established that doses of 70Gy or over are required for eradication of local disease while the highest radiation dose ever reported in a randomized dose escalation trial using conventionally fractionated radiation was 80Gy. The intermediate bound was set at 74Gy considering that dose escalation with 3D-CRT up to 74Gy was shown to achieved high control rates without an increase in treatment morbidity as compared to 70Gy [19]. We have observed no difference on bPFS in our population regardless of radiation dose. This may be explain by the fact that the majority of our patients (59.9%) had a baseline PSA < 10 ng/ml whereas the advantage of high dose radiotherapy seems to be more important in patients with PSA > 10-15ng/ml [3, 6]. We acknowledge the fact that for patients in oldest cohort it is likely possible to have counted PSA recurrences earlier than for those who were analyzed using Phoenix definition of PSA relapse. However, less than 20 pts (roughly 7%) completed treatment 2 years short from publication of Phoenix criteria. The unbalance in ADT use was accounted in both uni- and multivariate analysis as ADT may overcast the impact of radiation dose.

Secondly, the analytical part of our study was focused on intermediate doses (74Gy group), which are often used in clinical practice but nowadays often overlooked in the literature. Elderly pts may experience more severe radiation induced toxicity in contrast with younger men thus more modest dose radiation schedules such as those used in our series (74Gy) may be an alternative option. In a series of 132 pts treated with prostate-only radiotherapy, 35% of men over 75 years developed acute grade ≥2 rectal toxicity compared to 15% of pts under 75 years. Indeed, Sundar and colleagues [20] found that age above 75 years increases by 3.8 times the risk of rectal toxicity (OR 95% CI = 1.06–13.5). Furthermore, it seems that elderly pts with prostate cancer prefer lower radiation doses over efficacy [21].

Thirdly, we noted excellent rates of overall late grade ≥2 rectal toxicity which sustains other observations showing that IMRT is associated with decreased rectal toxicity compared to 3D-CRT [22]. We have applied the same PTV margins for 70 and 80Gy –groups as those used with 3D-CRT in the GETUG 06 protocol. We obtained a low rate of 5-year Grade ≥2 toxicity (6.2%) which can be thus considered as indirect evidence of the effectiveness of GETUG 06 constraints on rectal wall [3]. The actuarial grade ≥2 toxicity rates issued from investigations are in line with ours: 7.4% at 2 years in the series by Jereszek-Fossa et al. using 3D conformal two-dynamic arc therapy (3D-ART) to deliver a median dose of 76 Gy [23], 11% at 2 years according to De Meeleer et al. when using IMRT from 74 to 76Gy [24] and peak grade ≥2 toxicity of 10.9% for Vora et al. when delivering a median radiation dose of 75.6Gy [25]. The largest study of the Memorial Sloan Kettering Cancer Center (MSKCC) with high-dose IMRT (86.4Gy) showed 7-year actuarial rates of 4.4% Grade ≥2 toxicity. The PTV margins were basically identical to ours, 10mm in all directions except 6 mm posteriorly [26]. Due to a small number of adverse events (15 grade ≥2 toxicities) and the lack of some information on demographics and comorbidities, we failed to show that age, past medical history of pelvic or abdominal surgery, hemorrhoids, anticoagulation treatment are predictive of late rectal toxicity as some studies have suggested it [27–30]. No patient with ADT >6 months in our cohort had a rectal toxicity ≥ grade 2. The impact of ADT on late gastrointestinal toxicity is still controversial, previous reports showing no significant differences in late toxicity rates with or without the addition of ADT to radiation [31].

Fourthly, we have tried to identify patient and radiation related risk factors correlated with late urinary toxicity. So far, this has been rarely examined and very little is known on the topic. When designing this study authors had in mind to investigate dose constraints for urinary bladder with moderate dose IMRT (74Gy). The premise was again the study conducted on the behalf of the French GETUG who showed that dose escalation from 70 Gy to 80 Gy with 3D-CRT is more frequently associated with grade ≥2 late urinary toxicity when maximum radiation dose was above 75Gy (*p* = 0.0064) and 50% of the bladder received not more than 44.7 Gy (*p* = 0.04) [32]. The GETUG 06 cutoff values were used as dose constraints for bladder wall, having in mind that planning margins were identical. Volume receiving 65Gy (V65) ≤25% was equally defined as an additional constraint for the bladder wall (Table 1, column 2). Similar to the GETUG 06 results, we were able to show that bladder wall high dose spots, quantified as Dmax ≥ 74 Gy and D2% ≥ 73Gy are related to late urinary toxicity indicating that the maximal dose with IMRT seems to be important in determining toxicity.

The 5-year incidence of grade ≥2 late urinary toxicity was found to be of 24.9%, slightly higher to those reported in the literature ranging from 12 to 22% at 3 years [25, 33, 34]. Excellent outcomes were reported by Jereszek- Fossa [23] who found actuarial rates of 8.5% of grade 2 bladder toxicity at 2-years, which is probably due to a shorter follow-up period (23.5 months). Mild genitourinary toxicity is known to develop even into the second decade after radiotherapy although severe toxicity seems to be rare [35].

We also observed significant differences in grade ≥2 urinary toxicity between pts with a baseline IPSS ≥8 and those with an IPSS ≤8. To our knowledge, few studies have explored this potential predictor for radiation toxicity. In the series by Malik R et al., the 4-year freedom from grade ≥2 toxicity was significantly different in men with baseline IPSS ≥15 vs. ≤14 (38% vs. 64%, *p* < 0.0001). Grade 3 side effects were equality more common in pts with an IPSS ≥15 but this difference was not statistically significant [36]. Recent data from an analysis of 1002 pts treated with high-dose IMRT (86.4Gy) demonstrated that baseline IPSS >15 vs. ≤15 was an independent predictor of grade ≥2 late toxicity [26]. Hoffman et al. demonstrated that men with larger prostate pre-treatment volume have an increased risk of late toxicity [37], knowing that larger prostate volumes are correlated with higher baseline IPSS [38]. Nevertheless, no clear threshold value could be identified from the published literature.

Because this study is retrospective, it is subject to bias in patient selection. Further limitation of this study was the fact that our toxicity grading was limited to case report forms performed during consultations and follow-up visits without patient based self-assessment questionnaires. Combing both seems to be more accurate in quantifying the true incidence of radiation induce toxicity [39]. Furthermore, the use of ADT has been reported to increase urinary toxicity and thus induce biases in toxicity analysis. Like mention before, the unbalanced in ADT use between our groups was accounted in the multivariate analysis.

## Conclusion

Delivering 80Gy for a CaP with IMRT increases the risk of grade ≥2 overall rectal toxicity as compared to ≤74Gy but rates are low when compared to 3D-CRT using the same safety margins. Predictors of overall grade ≥2 urinary toxicity were identified for 74Gy. Image-guidance IMRT (IG-IMRT) with either implanted fiducial markers or cone beam computed tomography was implemented in our department.

## Abbreviations

3D-CRT: Three-dimensional conformal radiotherapy
ADT: Androgen deprivation therapy
bPFS: Biochemical progression free survival
CPHM: Cox proportional hazard model
CTCAE: Common Terminology Criteria for Adverse Events
Dmax: Maximum dose
D2%: Near-maximum dose
GETUG: French Study Group on Urogenital Tumors
HIFU: High intensity frequency ultrasound
IMRT: Intensity-modulated radiotherapy
IPSS: International Prostate Symptom Score
PSAn: PSA nadir
Pts: Patients
PTV: Planning target volume
SIB: Simultaneous integrated boost
tPSAn: Time to PSA nadir

## References

[1] Symon Z, Griffith K, McLaughlin PW, Sullivan M, Sandler HM (2003). Dose escalation for localized prostate cancer: Substantial benefit observed with 3D conformal therapy. Int J Radiat Oncol Biol Phys.

[2] Dearnaley DP, Jovic G, Syndikus I, Khoo V, Cowan R, Graham JD (2014). Escalated-dose versus control-dose conformal radiotherapy for prostate cancer: Long-term results from the MRC RT01 randomised controlled trial. Lancet Oncol.

[3] Beckendorf V, Guerif S, Le Prisé E, Cosset JM, Bougnoux A, Chauvet B (2011). 70 Gy versus 80 Gy in localized prostate cancer: 5-year results of GETUG 06 randomized trial. Int J Radiat Oncol Biol Phys.

[4] Kuban D, Tucker SL, Dong L, Starkschall G, Huang EH, Cheung MR (2008). Long-term results of the M. D. Anderson randomized dose-escalation trial for prostate cancer. Int J Radiat Oncol Biol Phys.

[5] Zietman AL, DeSilvio ML, Slater JD, Rossi CJ, Miller DW, Adams JA (2005). Comparison of conventional-dose vs high-dose conformal radiation therapy in clinically localized adenocarcinoma of the prostate. JAMA.

[6] Heemsbergen WD, Al-Mamgani A, Slot A, Dielwart MFH, Lebesque JV (2014). Long-term results of the Dutch randomized prostate cancer trial: Impact of dose-escalation on local, biochemical, clinical failure, and survival. Radiother Oncol.

[7] Sathya JR, Davis IR, Julian J, Guo Q, Daya D, Dayes IS (2005). Randomized trial comparing iridium implant plus external-beam radiation therapy with external-beam radiation therapy alone in node-negative locally advanced cancer of the prostate. J Clin Oncol.

[8] Zelefsky MJ, Yamada Y, Fuks Z, Zhang Z, Hunt M, Cahlon O (2008). Long-term results of conformal radiotherapy for prostate cancer: impact of dose escalation on biochemical tumor control and distant metastases-free survival outcomes. Int J Radiat Oncol Biol Phys.

[9] Ohri N, Dicker AP, Showalter TN (2012). Late toxicity rates following definitive radiotherapy for prostate cancer. Can J Urol.

[10] Michalski JM, Bae K, Roach M, Markoe AM, Sandler HM, Ryu J (2010). Long-term toxicity following 3D conformal radiation therapy for prostate cancer from the RTOG 9406 phase I/II dose escalation study. Int J Radiat Oncol Biol Phys.

[11] Syndikus I, Morgan RC, Sydes MR, Graham JD, Dearnaley DP (2010). Late gastrointestinal toxicity after dose-escalated conformal radiotherapy for early prostate cancer: results from the UK Medical Research Council RT01 trial (ISRCTN47772397). Int J Radiat Oncol Biol Phys.

[12] Fransson P, Bergström P, Löfroth P-O, Widmark A (2006). Five-year prospective patient evaluation of bladder and bowel symptoms after dose-escalated radiotherapy for prostate cancer with the BeamCath technique. Int J Radiat Oncol Biol Phys.

[13] Zelefsky MJ, Yamada Y, Kollmeier MA, Shippy AM, Nedelka MA (2008). Long-term outcome following three-dimensional conformal/intensity-modulated external-beam radiotherapy for clinical stage T3 prostate cancer. Eur Urol.

[14] D’Amico AV, Whittington R, Malkowicz SB, Schultz D, Blank K, Broderick GA (1998). Biochemical outcome after radical prostatectomy, external beam radiation therapy, or interstitial radiation therapy for clinically localized prostate cancer. JAMA.

[15] Roach M, Hanks G, Thames H, Schellhammer P, Shipley WU, Sokol GH, Sandler H (2006). Defining biochemical failure following radiotherapy with or without hormonal therapy in men with clinically localized prostate cancer: recommendations of the RTOG-ASTRO Phoenix Consensus Conference. Int J Radiat Oncol Biol Phys.

[16] American Society for Therapeutic Radiology and Oncology (1997). consensus statement:guidelines for PSA following radiation therapy. Int J Radiat Oncol Biol Phys.

[17] Barry MJ, O’Leary MP, Bruskewitz RC, Holtgrewe HL, Mebust WK, Cockett ATFFJJ (1992). The American urological association symptom index for benign prostatic hyperplasia. The measurement commettee of the American Urological Association. J Urol.

[18] Sauerbrei W (1999). The use of resampling methods to simplify regression models in medical statistics. J R Stat Soc Ser C Appl Stat.

[19] Goldner G, Bombosch V, Geinitz H, Becker G, Wachter S, Glocker S (2009). Moderate risk-adapted dose escalation with three dimensional conformal radiotherapy of localised prostate cancer from 70 to 74 Gy. Strahlenther Onkol.

[20] Sundar S, Walker G, Pascoe A (2016). Should elderly prostate cancer patients with comorbidity be spared from prostate radiation dose escalation?. Clin Oncol (R Coll Radiol).

[21] van Tol-Geerdink JJ, Stalmeier PFM, van Lin ENJT, Schimmel EC, Huizenga H, van Daal WAJ (2006). Do patients with localized prostate cancer treatment really want more aggressive treatment?. J Clin Oncol.

[22] Wortel RC, Incrocci L, Pos FJ, van der Heide UA, Lebesque JV, Aluwini S (2016). Late side effects after image guided intensity modulated radiation therapy compared to 3D-conformal radiation therapy for prostate cancer: results from 2 prospective cohorts. Int J Radiat Oncol Biol Phys.

[23] Jereczek-Fossa BA, Vavassori A, Fodor C, Santoro L, Zerini D, Cattani F (2008). Dose escalation for prostate cancer using the three-dimensional conformal dynamic Arc technique: analysis of 542 consecutive patients. Int J Radiat Oncol Biol Phys.

[24] De Meerleer GO, Fonteyne VH, Vakaet L, Villeirs GM, Denoyette L, Verbaeys A (2007). Intensity-modulated radiation therapy for prostate cancer: late morbidity and results on biochemical control. Radiother Oncol.

[25] Vora SA, Wong WW, Schild SE, Ezzell GA, Andrews PE, Ferrigni RG (2013). Outcome and toxicity for patients treated with intensity modulated radiation therapy for localized prostate cancer. J Urol.

[26] Spratt DE, Pei X, Yamada J, Kollmeier MA, Cox B, Zelefsky MJ (2013). Long-term survival and toxicity in patients treated with high-dose intensity modulated radiation therapy for localized prostate cancer. Int J Radiat Oncol Biol Phys.

[27] Fiorino C, Fellin G, Rancati T, Vavassori V, Bianchi C, Borca VC (2008). Clinical and dosimetric predictors of late rectal syndrome after 3D-CRT for localized prostate cancer: preliminary results of a multicenter prospective study. Int J Radiat Oncol Biol Phys.

[28] Pederson AW, Fricano J, Correa D, Pelizzari CA, Liauw SL (2012). Late toxicity after intensity-modulated radiation therapy for localized prostate cancer: An exploration of dose-volume histogram parameters to limit genitourinary and gastrointestinal toxicity. Int J Radiat Oncol Biol Phys.

[29] Valdagni R, Vavassori V, Rancati T, Fellin G, Baccolini M, Bianchi C (2012). Increasing the risk of late rectal bleeding after high-dose radiotherapy for prostate cancer: The case of previous abdominal surgery. Results from a prospective trial. Radiother Oncol.

[30] Choe KS, Jani AB, Liauw SL (2010). External beam radiotherapy for prostate cancer patients on anticoagulation therapy: How significant is the bleeding toxicity?. Int J Radiat Oncol Biol Phys.

[31] Jani AB, Gratzle J (2005). Late radiotherapy toxicity after prostate cancer treatment: influence of hormonal therapy. Urology.

[32] Beckendorf V, Guerif S, Le Prise E, Cossett JM, Le Floch O, Chauvet B (2007). 2210 Late Toxicity in the GETUG 06 Randomized Trial Comparing 70 Gy and 80 Gy for Localized Prostate Cancer. Int J Radiat Oncol Biol Phys.

[33] Alicikus ZA, Yamada Y, Zhang Z, Pei X, Hunt M, Kollmeier M (2011). Ten-year outcomes of high-dose, intensity-modulated radiotherapy for localized prostate cancer. Cancer.

[34] Fonteyne V, Villeirs G, Lumen N, De Meerleer G (2009). Urinary toxicity after high dose intensity modulated radiotherapy as primary therapy for prostate cancer. Radiother Oncol.

[35] Gardner BG, Zietman AL, Shipley WU, Skowronski UE, McManus P (2002). Late normal tissue sequelae in the second decade after high dose radiation therapy with combined photons and conformal protons for locally advanced prostate cancer. J Urol.

[36] Malik R, Jani AB, Liauw SL (2011). External beam radiotherapy for prostate cancer: Urinary outcomes for men with high International Prostate Symptom Scores (IPSS). Int J Radiat Oncol Biol Phys.

[37] Hoffman KE, Voong KR, Pugh TJ, Skinner H, Levy LB, Takiar V (2014). Risk of Late toxicity in men receiving dose-escalated hypofractionated intensity modulated prostate radiation therapy: Results from a randomized trial. Int J Radiat Oncol Biol Phys.

[38] Narayanan R, Rambarran L, Kubicek G, Chevli KK, Duff M (2013). Effect of pretreatment prostate volume on urinary quality of life following intensity-modulated radiation therapy for localized prostate cancer. Res Rep Urol.

[39] Goldner G, Wachter-Gerstner N, Wachter S, Dieckmann K, Janda M, Pötter R (2003). Acute side effects during 3-D-planned conformal radiotherapy of prostate cancer. Differences between patient’s self-reported questionnaire and the corresponding doctor’s report. Strahlenther Onkol.

